# Characterization, Density and In Vitro Controlled Release Properties of Mimosa (*Acacia mearnsii*) Tannin Encapsulated in Palm and Sunflower Oils

**DOI:** 10.3390/ani11102919

**Published:** 2021-10-09

**Authors:** Shehu Lurwanu Ibrahim, Abubeker Hassen

**Affiliations:** Department of Animal Science, University of Pretoria, Private Bag X20, Hatfield 0028, South Africa; abumubarak480@gmail.com

**Keywords:** particle density, gastrointestinal tract, microencapsulation, mimosa tannin, morphology, palm oil, sunflower oil, release kinetics

## Abstract

**Simple Summary:**

The utilization of tannin in mitigating enteric methane suffers a setback in terms of dietary intake and digestibility because of the tannin’s astringency and instability in the gastrointestinal tract. Microencapsulation of tannin using lipids could mask its bitter taste and ensure its controlled release at the target site. This study aimed to encapsulate *Acacia mearnsii* tannin extract with palm and sunflower oils, and to evaluate the efficacy of the encapsulated tannins with regards to encapsulation efficiency, density, and release of tannin in media, simulating the rumen, abomasum and the small intestine. Mimosa tannin was encapsulated in palm oil or sunflower oil using a double emulsion method. The findings showed that encapsulated mimosa tannins in the palm oil and sunflower oil had high encapsulation efficiencies with smaller sizes and were lower in density compared to the unencapsulated mimosa tannin. The amount of tannins released by the unencapsulated tannin after 24 h in rumen (94%), abomasum (92%) and small intestine (96%) simulated buffers, were reduced to 24%, 21% and 19%, respectively, for the sunflower oil microparticle and 18%, 20% and 16%, respectively, for the palm oil microparticle in the same buffers and periods. Palm oil and sunflower oil successfully encapsulated the mimosa tannin and controlled its release in the gastrointestinal tract simulated media without compromising rumen fermentation.

**Abstract:**

Tannin has gained wider acceptance as a dietary supplement in contemporary animal nutrition investigations because of its potential to reduce enteric methane emission. However, a major drawback to dietary tannin intake is the bitter taste and instability in the gastrointestinal tract (GIT). The utilization of fats as coating materials will ensure appropriate masking of the tannin’s aversive taste and its delivery to the target site. The aims of this study were to encapsulate mimosa tannin with palm oil or sunflower oil, and to assess the microcapsules in terms of encapsulation efficiency, morphology, density, and in vitro release of tannin in media simulating the rumen (pH 5.6), abomasum (pH 2.9) and small intestine (pH 7.4). The microencapsulation of mimosa tannin in palm or sunflower oils was accomplished using a double emulsion technique. The results revealed that encapsulated mimosa tannins in palm oil (EMT^P^) and sunflower oil (EMT^S^) had high yields (59% vs. 58%) and encapsulation efficiencies (70% vs. 68%), respectively. Compared to unencapsulated mimosa tannin (UMT), the morphology showed that the encapsulated tannins were smaller in size and spherical in shape. The UMT had (*p* < 0.01) higher particle density (1.44 g/cm^3^) compared to 1.22 g/cm^3^ and 1.21 g/cm^3^ for the EMT^S^ and EMT^P^, respectively. The proportion of tannins released by the UMT after 24 h in the rumen (94%), abomasum (92%) and small intestine (96%) simulated buffers, reduced (*p* < 0.01) to 24%, 21% and 19% for the EMT^S^ and 18%, 20% and 16% for the EMT^P^ in similar media and timeframe. The release kinetics for the encapsulated tannins was slow and steady, thus, best fitted by the Higuchi model while the UMT dissolved quickly, hence, only fitted to a First order model. Sequential tannin release also indicated that the EMT^S^ and EMT^P^ were stable across the GIT. It was concluded that the microencapsulation of mimosa tannin in palm or sunflower oils stabilized tannins release in the GIT simulated buffers with the potential to modify rumen fermentation. Further studies should be conducted on the palm and sunflower oils microcapsules’ lipid stability, fatty acid transfer rate in the GIT and antioxidant properties of the encapsulated tannins.

## 1. Introduction

The utilization of tannins as feed supplements in recent ruminant nutrition studies is linked to their positive roles in modulating rumen fermentation, methane (CH_4_) emission and protein metabolism [1]. Enteric CH_4_ is one of the by-products of anaerobic fermentation of structural carbohydrates in the rumen which has a potential global warming impact twenty-five times greater than that of carbon dioxide [2]. Several studies have showed that condensed tannins are capable of mitigating CH_4_ emissions either directly by interfering with the proliferation and activities of methanogens or indirectly through the reduction in fiber degradation and hindrance of protozoa activities [3,4,5]. In addition, condensed tannins have the ability to bind dietary proteins at the normal rumen pH, thereby increasing amino acid absorption in the small intestine [6,7].

Among the condensed tannins of the rich leguminous trees mostly consumed by ruminants in the tropics [8], *Acacia mearnsii,* popularly known as mimosa tannin [9], is considered as the most widely spread and highly invasive alien species in South Africa, covering an area of over 2.5 million hectares [10], and more than 130,000 ha of commercial plantations [11]. Numerous studies have revealed that Mimosa tannin has the potential to reduce enteric CH_4_ and ammonia nitrogen (NH_4_-N) production while enhancing dietary protein by-pass. For instance, when an extract of mimosa tannin was fed to cows, CH_4_ and urine nitrogen were reduced by up to 29% and 9.3%, respectively [12]. Likewise, in sheep, urine nitrogen dropped by 59% and CH4 by 13% [13]. Additionally, *A. mearnsii* tannins increased propionate levels beyond 100 g/kg of dry matter (DM) against the acetate [14].

However, the oral administration of tannins suffers some drawbacks in terms of dietary intake and digestibility, due to the bitter taste largely attributed to the tannins’ negative reaction with salivary proteins [15], as well as their instability and binding nature in the gastrointestinal tract (GIT) [16]. Furthermore, intake and digestibility of the tannins have been found to reduce when condensed tannins are fed above 50 g/kg of DM [17,18]. Some studies such as that of Priolo et al. [19], reported a 48 g reduction in average daily gain in sheep while Bhatta et al. [20] observed a decrease of 30% in nitrogen retention and an increase in energy loss up to 45% in goats supplemented with high amounts of condensed tannins. In addition, Grainger et al. [12] recorded a 29.7% reduction in milk yield when a large quantity of mimosa tannin extract was fed to cows. Therefore, there is a need for the development of a technique that will ensure the appropriate masking of tannins’ aversive taste and their sustained release into the GIT without compromising normal rumen function.

Various encapsulation technologies have been developed by feed and pharmaceutical industries to conceal the aversive tastes of many bioactive compounds, to improve their stability and control their release to target site of digestion without any adverse effects [21,22]. Bakry et al. [23] defined encapsulation as a method of building an efficient barrier between the active ingredients and coating material to prevent any form of chemical and physical reactions, and to sustain the biological, functional, and physicochemical characteristics of the active ingredient. Numerous microencapsulation methods were adopted such as spray-drying, spray-cooling, spray-chilling, freeze-drying, centrifugal suspension separation, inclusion complexation and coacervation using various wall materials [24]. The best coating material for use in masking the bitter taste of tannins should be inexpensive, tasteless, have low viscosity, be a good film former, have good emulsifying properties and be able to safeguard the bioactive compound up to the target site [25,26].

However, the feed industry is faced with the challenges of selecting appropriate wall materials that are available, inexpensive, and safe [27], which affect the practicability of encapsulation technology in animal nutrition. In southern Africa, sunflower oil and palm oil are found in abundance and could serve as suitable wall materials for tannin encapsulation due to their desirable aroma [23], good emulsifying properties and low viscosity [28]. A review by Eckard et al. [29] reported a significant reduction in CH_4_ when sunflower oil was supplemented in the diets of ruminants. In addition, in vitro CH_4_ volume dropped when palm oil was adopted as a coating material to encapsulate *A. mearnsii* extract using a solid-in-oil-water (S/O/W) technique [30]. However, literature on using lipids such as sunflower and palm oils as coating materials to mask the tannins’ bitter taste and ensure their controlled release to the target sites of function in the GIT is still scarce. The objectives of the present study, therefore, are to encapsulate mimosa tannin with sunflower and palm oils using the double emulsion method, and to evaluate the sunflower and palm oils’ microparticles in terms of their encapsulation efficiency, morphology, density, and in vitro release rate in various media simulating the GIT.

## 2. Materials and Methods

### 2.1. Study Area

The experiment was conducted in the Department of Animal Sciences, University of Pretoria, South Africa. The location lies at a latitude 25°44′30″ south and longitude 28°15′30′′ east at an altitude of 1360 m above sea level [31]. This research was approved by the Animal Ethics Committee of the University of Pretoria (Ref No: EC075-17).

### 2.2. Materials

The mimosa (*Acacia mearnsii*) tannin extract used in this study was gifted by the UCL company Pty (Ltd), Dalton, South Africa. The extract was reported to have been obtained from the bark of the *A. mearnsii* tree following hot water extraction processes at specific temperature, pressure, and time. The water was removed using a vacuum evaporator and the extract was air-dried into fine powder, packaged, and refrigerated before use [32]. The palm and sunflower oils were purchased from Pick ‘n’ Pay grocery, Hatfield, Pretoria. The emulsifiers (Span80, Tween80) and dichloromethane (DCM) were procured from Sigma-Aldrich, St. Louis, MO, USA. Filter bags (F57 fiber) were purchased from ANKOM Technology, New York, NY, USA. All other reagents utilized were of analytical grade and sourced from Sigma-Aldrich, Johannesburg, South Africa.

### 2.3. Characterization of Mimosa Tannin

Mimosa tannin powder (0.2 g) was added in 25 mL volume glass beakers containing aqueous acetone (10 mL) and dissolved for 20 min in an ultrasonic bath. The solution was transferred into tubes and centrifuged in a refrigerated centrifuge at 2500 rpm for 15 min. and the supernatant collected and kept in ice blocks. The concentrations of total phenol, non-tannin phenols and total tannins in the extracts were determined using the Folin–Ciocalteu method [33], as a tannic acid equivalent, while condensed tannin content was analyzed as a leucocyanidin equivalent using the Butanol–HCl method [34]. The proportion of hydrolysable tannin was estimated by the differences between the total tannins and condensed tannins [35].

### 2.4. Microencapsulation of Mimosa Tannin

Mimosa tannin was encapsulated with either palm oil or sunflower oil using the S/O/W technique described by Adejoro et al. [30] with little modification. The water (W) solution was first prepared in a 500 mL beaker by adding 300 mL distilled water comprising 1% (*w*/*v*) Tween80 emulsifier. The mixture was then homogenized using an iron rod homogenizer (PRO400DS, Pro Scientific Inc., Oxford, CT 06478, USA) set at 20,000 revolutions per minute (rpm) for 3 min until the solution foamed. The solid-in-oil (S/O) solution was simultaneously prepared by adding 8.5 g of mimosa tannin powder into a 100 mL beaker containing 30 mL palm or sunflower oil solution in DCM (50 mg/mL) mixed with 0.5% (*w*/*v*) Span80 as a surfactant then stirred thoroughly for 2 min using a magnetic stirrer set at 400 rpm. The S/O solution was subsequently added to the W solution and homogenized for 3 min at 20,000 rpm to form the final S/O/W mixture. The mixture was stirred for three hours using a magnetic stirring plate set at 800 rpm to completely evaporate the DCM. The palm and sunflower oil microcapsules produced were squeezed through a four-fold layer of cleaned cheese cloth, rinsed with about 100 mL distilled water, transferred into the aluminum container, and freeze-dried for 5 days. The encapsulated mimosa tannins in palm oil (EMT^P^) or sunflower oil (EMT^S^) were collected, ground to powder and refrigerated before analysis.

### 2.5. Optimization of Mimosa Tannin Microcapsules

#### 2.5.1. Encapsulation Efficiency and Tannin Yield

The encapsulation efficiency (Ee) and tannin yield of sunflower and palm oil microcapsules were determined according to the procedure of Adejoro et al. [30], with slight changes. The powdered samples (0.1 g) of EMT^P^ and EMT^S^ were separately weighed into a beaker containing DCM (20 mL), and heated in an ultrasonic bath for 10 min to disband the lipid coatings. The solutions were centrifuged at 2500 rpm for 10 min and the tannin pellets were collected and dissolved in 70% aqueous acetone (20 mL) to reconstitute the extracts. The concentration of the actual loaded tannin (LT_a_) was determined using a spectrophotometer absorbance at 725 nm. The Ee of the EMT^P^ and EMT^S^ were estimated from the Equation (1) below:

(1) $$\mathrm{Ee}\;\left(\%\right)=\frac{{\mathrm{LT}}_{a}}{{\mathrm{LT}}_{t}}\times 100$$

where Ee = encapsulation efficiency, LT_a_ = actual loaded tannin (%, *w*/*w*) in the oil microcapsules and LT_t_ = theoretical loaded tannin (i.e., the amount of mimosa tannin added during encapsulation).

The mimosa tannin encapsulation yield (%) was estimated from the total tannin content obtained in the EMT^P^ and EMT^S^ microcapsules and the total amount of tannin initially added as shown in Equation (2):

(2) $$\mathrm{Yield}\;\left(\%\right)=\frac{\mathrm{amount}\;\mathrm{of}\;\mathrm{tannin}\;\mathrm{obtained}\;\left(g\right)}{\mathrm{total}\;\mathrm{tannin}\;\mathrm{added}\;\left(g\right)}\times 100$$

#### 2.5.2. Scanning Electron Microscopy

The morphology of the EMT^P^ and EMT^S^ compared to the unencapsulated mimosa tannin (UMT) were determined using a scanning electron microscope (SEM) following the procedure of Taylor et al. [36] with slight modifications. The powdered samples (UMT, EMT^P^ and EMT^S^) were prepared separately and smeared with carbon, then mounted to the stubs of the SEM (JEOL, JSM-840 Tokyo, Japan) using adhesive tape and viewed at 20 kV.

#### 2.5.3. Microparticle Density

The three mimosa tannin samples (UMT, EMT^P^ and EMT^S^) were evaluated for particle density using a Gas Pycnometer (AccuPyc II 1340 Micromeritics Instr. Corp. Norcross, GA, USA). The powdered samples (52 g) were separately transferred and sealed into the vessel of the Pycnometer. The vessel used an automated pressure determining technique to detect the pressure change resulting from the displacement of Helium gas by the tannin samples. Helium was used as the choice gas medium because it is small enough to penetrate virtually all connected pores within a sample. The volume determined was finally divided into sample weights and the microparticle densities of the UMT, EMT^P^ and EMT^S^ were recorded.

### 2.6. In Vitro Release of Tannin from Oil Microcapsules

The in vitro release properties of the UMT, EMT^P^ and EMT^S^ were evaluated following the procedure of Adejoro et al. [30] with little change. Three different buffer solutions were prepared: an acetate buffer (pH 5.6), citrate buffer (pH 2.9) and phosphate buffer (pH 7.4) simulating the rumen, abomasum, and small intestine pH, respectively [37,38] Samples (100 mg) from each of the three mimosa tannin treatments (UMT, EMT^P^ and EMT^S^) were separately weighed into 25 μm porosity filter bags (F57; ANKOM) and suspended in three separate bottles containing 50 mL solutions of acetate, citrate and phosphate buffers. The bottles were placed in an incubator shaker and rotated at 50 rpm at 39 °C. The solutions were sampled (2 mL) in triplicates at intervals as follows: 1, 2, 4, 8, and 24 h of incubation. The original volume of the media was maintained by the replacement of a 2 mL fresh buffer immediately after each sampling. The buffer samples collected were frozen immediately for latter analysis. The proportion of mimosa tannins released by the UMT, EMT^P^ and EMT^S^ into the three different pH media simulating the GIT at 1, 2, 4, 8, and 24 h were assessed by spectrophotometer absorbance at 725 nm.

The mimosa tannin release kinetics were assessed using the Zero order, First order and Higuchi model (Equations 3–5, respectively) to determine the best model for tannins released by UMT, EMT^P^ and EMT^S^ microcapsules in the rumen, abomasum and small intestinal simulated buffer as described by Adejoro et al. [30] and Tolve et al. [39]:

(3) $$Q_{t}=Q_{0}+{\;Q}_{0}t$$

(4) $${\mathrm{logQ}}_{t}={\mathrm{logQ}}_{0}-{\;Q}_{1}t$$

(5) $$Q_{t}=Q_{0}-{\;Q}_{H}t^{1/2}$$

where k_0_ = Zero-order rate constant; t = time; Q_t_ = released concentration of mimosa tannin at time t; Q_0_ = initial concentration of tannins within solutions (usually Q_0_ = 0); k_1_ = First order rate constant and k_H_ = Higuchi dissolution constant.

According to Tolve et al. [39], the Zero order kinetic model describes the phenomenon of slow-release, in a shell that does not disintegrate easily and generally applicable to poorly soluble compounds. In First order kinetics, the dissolution of the bioactive compound, which is usually soluble in water and entrapped in a porous shell material, is proportional to its concentration. The Higuchi model refers to release kinetics involving both diffusion and dissolution.

Another experiment was carried out to examine the sequential tannin release at 24 h in a rumen simulated medium (pH 5.6), and at 8 h each in abomasum (pH 2.9) and small intestine (pH 7.4) simulated media. The three mimosa tannin treatments (UMT, EMT^P^ and EMT^S^) were weighed (100 mg) in filter bags and suspended in bottles containing acetate buffer (50 mL). The bottles were placed in shaker incubator and rotated at 50 rpm at 39 °C. The solutions were sampled after 24 h and the filter bags containing the unencapsulated and encapsulated tannin residues were rinsed with water and subsequently transferred to bottles containing citrate buffer inside incubator shaker set at 50 rpm and 39 °C. The solutions were sampled after 8 h incubation and the bags containing residue samples were rinsed with water and then suspended in the bottles containing phosphate buffer. The bottles were similarly incubated at 50 rpm and 39 °C and then sampled after 8 h. Following each incubation period, extracts (2 mL) were sampled in triplicate and stored inside vials at −20 °C before analysis. Three independent incubation cycles was carried out across the three buffer media. The proportion of mimosa tannins released by the UMT, EMT^P^ and EMT^S^ sequentially after 24 h of incubation in the respective buffer simulating the rumen, abomasum and small intestine were determined from a spectrophotometer absorbance at 725 nm.

### 2.7. Statistical Analysis

All data were coded in a Microsoft Excel (Microsoft Corp. Redmond, WA, USA) spread sheet and analyzed for variance using SAS version 9.4 (SAS Institute Inc., Carry, NC, USA). Data on encapsulation efficiency and tannin yield, tannin microparticle density and tannin release rate for the UMT, EMT^P^ and EMT^S^ at various pH were subjected to one-way ANOVA. Significantly different means were separated using the least significant difference (LSD) and differences reported at 5% or a 1% level of probability where applicable.

## 3. Results and Discussion

### 3.1. Characterization of Mimosa Tannins

Prior to the encapsulation process, mimosa tannin was characterized to ascertain its total phenol, non-tannin phenol, total tannin, condensed tannin and hydrolysable tannin concentrations. The extract comprised of tannin phenol (699.3 g kg^−1^ DM), non-tannin phenol (32.3 g kg^−1^ DM), total tannin (677.6 g kg^−1^ DM), hydrolysable tannin (463.8 g kg^−1^ DM) and condensed tannin (221.7 g kg^−1^ DM). In corroboration with this finding, several studies reported comparable results [30,32,40,41,42] for the mimosa tannin characterization.

### 3.2. Optimization of Mimosa Tannins’ Microcapsules

The present study did not observe any significant differences between the mimosa tannins encapsulated with palm oil and sunflower oil in terms of tannin yield and encapsulation efficiency. However, a good proportion of tannin yields (59% vs. 58%) and encapsulation efficiencies (70% vs. 68%) were obtained from the EMT^P^ and EMT^S^ microcapsules, respectively. According to Mehran et al. [43], an effective encapsulation technique ensures a high retention of the core materials and only low concentration is trapped on the surface. Moreover, encapsulation efficiency of lipid microcapsules has been shown to depend on the emulsifying properties and viscosity of the coating materials [23,28], as well as the tannin particle size [44] Although, the palm and sunflower microparticles did not differ statistically with regards to their encapsulation efficiency and tannin yield, nevertheless, the slightly higher Ee and yield for the EMT^P^ could be attributed to its superior viscosity compared to the EMT^S^. Davies [45] reported that palm oil had a viscosity of between 20 and 39.5 cP while, sunflower oil had between 17 and 29 cP. The higher encapsulation efficiency and tannin yield recorded in the current study compares favorably with the findings of Adejoro et al. [30] who reported tannin yield of 63% vs. 57% for acacia tannin encapsulated with palm oil and lard, and Ee of 80% vs. 69%, respectively. Tolve et al. [39] also obtained a comparable encapsulation efficiency of 76% in Quebracho condensed tannin encapsulated with gum arabic–maltodextrin using a spray dryer.

**Figure 1:**
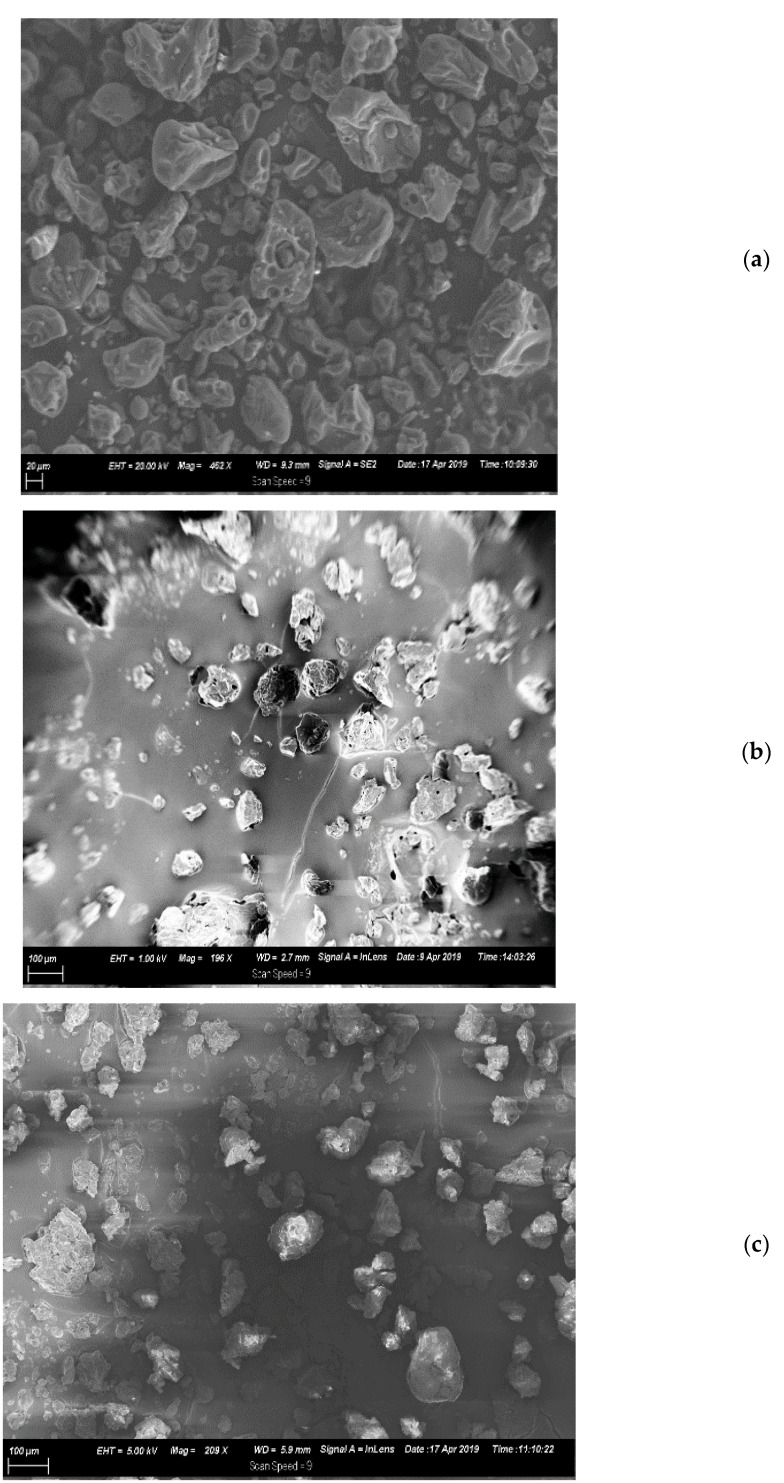
Morphology of *A. mearnsii* tannin particles viewed using SEM (**a**) unencapsulated mimosa tannin, UMT, (**b**) encapsulated mimosa tannin in palm oil, EMT^P^ and (**c**) encapsulated mimosa tannin in sunflower oil, EMT^S^.

Scanning electron microscopy (Figure 1a–c) indicated that EMT^P^ and EMT^S^ microparticles were smaller and more uniform in size and spherical in shape with a visible whitish oil color enveloping the tannin particles, while UMT morphology revealed tannin particles with bigger and more heterogeneous sizes, irregular shapes and a consistent dark brown colour. This showed that both palm and sunflower oils are good film formers which could be traced to their good emulsifying properties.

With respect to tannin particle density, the results showed that the UMT was heavier (1.44 g/cm^3^) than the EMT^S^ (1.22 g/cm^3^) and the EMT^P^ (1.21 g/cm^3^) (*p* < 0.01). However, there was no statistical difference between EMT^P^ and EMT^S^ microcapsules with regards to density. This showed that the unencapsulated mimosa tannin particles, being heavier, might sink to the bottom of the rumen and thus remain longer compared to the palm and sunflower oil microcapsules that, being lighter, could float in the middle of the rumen and hence quickly pass to the next compartment. Kaske and Engelhardt [46] reported that coarser and heavier feed particles had longer retention times in the rumen as well as other compartments.

### 3.3. Mimosa Tannin Release Rate Properties

Numerous studies have revealed the significance of feeding an optimum concentration of mimosa tannins to ruminants to enhance dietary protein utilization, reducing enteric methane production as well as improving the acetate: propionate ratio [12,13,14,47]. The quality of wall materials is associated with their capacity to preserve the bioactive compound and ensure its delivery to the target site of function [48,49]. Such quality can be assessed by carrying out a release rate trial using a GIT simulated buffer solution [47]. Table 1 presents the summary of in vitro tannin release for the UMT, EMT^P^ and EMT^S^ at predetermine hours in an acetate buffer (pH 5.6), citrate buffer (pH 2.9) and phosphate buffer (pH 7.4), simulating the rumen, abomasum, and small intestine, respectively. In the acetate buffer (pH 5.6), 68, 77, 81, 90 and 94% of UMT was released at 1, 2, 4, 8 and 24 h, respectively, and this was significantly higher (*p* < 0.01) compared to the 6.5, 10.3, 15, 19 and 24% of tannins released from the EMT^S^ and 5.2, 9.1, 12.3, 16 and 18.3% released from the EMT^P^ at 1, 2, 4, 8 and 24 h, respectively. There were no statistical effects on the amount of tannin released in a similar medium between the EMT^S^ and EMT^P^ at 1 and 2 h of incubation, however, the EMT^S^ had a higher tannin release rate from 4–24 h periods (*p* < 0.01).

In the citrate buffer (pH 2.9), a significantly higher proportion of mimosa tannins(69, 73, 79.3, 86.4 and 92.2%) was released compared to the 6.4, 9.7, 13.4, 17 and 20.4% of tannins released from the EMT^P^, and 6, 10, 14, 20 and 21.4% from EMT^S^ microcapsules at 1, 2, 4, 8 and 24 periods, respectively. Nevertheless, no differences in tanninrelease pattern were observed between the EMT^S^ and EMT^P^ at across the time periods (*p* > 0.05). In the phosphate buffer (pH 7.4), the UMT released higher proportion of tannins with 66, 71.4, 80.4, 87.4 and 96% released compared to 4.5, 7, 11, 14 and 19% released from the EMT^S^ microparticles and 4, 6, 9.3, 12 and 16% released from the EMT^P^, at 1, 2, 4, 8 and 24 h, respectively (*p* < 0.01). Tannin release rate was significantly different between the EMT^S^ and EMT^P^ only at 2 h of incubation across the acetate, citrate and phosphate buffer media. The significantly higher in vitro release rate of tannin from the UMT compared to those of the EMT^S^ and EMT^P^ across the incubation periods and buffer media could be linked to the entrapment of the tannin within the lipid wall matrices of the microcapsules. In addition, the EMT^S^ had a higher release rate than the EMT^P^, indicating that the microparticle with a higher Ee released lower tannins and vice versa. This finding is corroborated by the report of Adejoro et al. [30] who obtained a 20%, 34% and 25% tannin release from acacia encapsulated with lard and 19%, 30% and 22% from tannin encapsulated with palm oil in acetate, phosphate and HCl media, respectively, after 24 h incubation.

**Figure 2:**
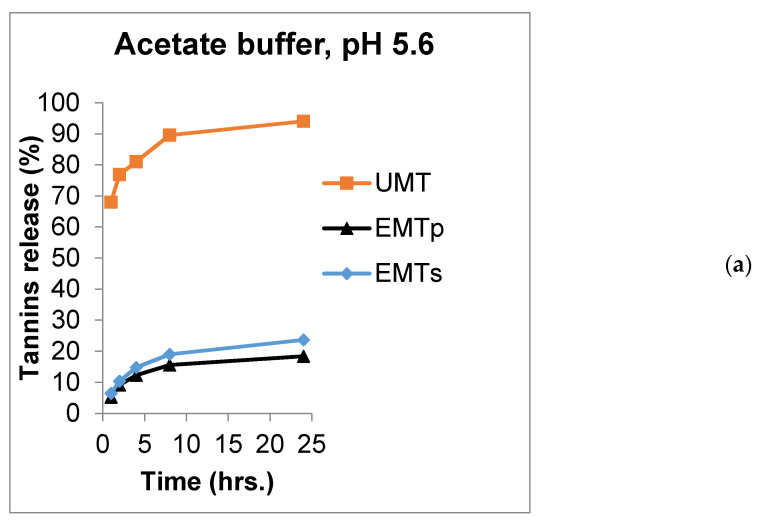

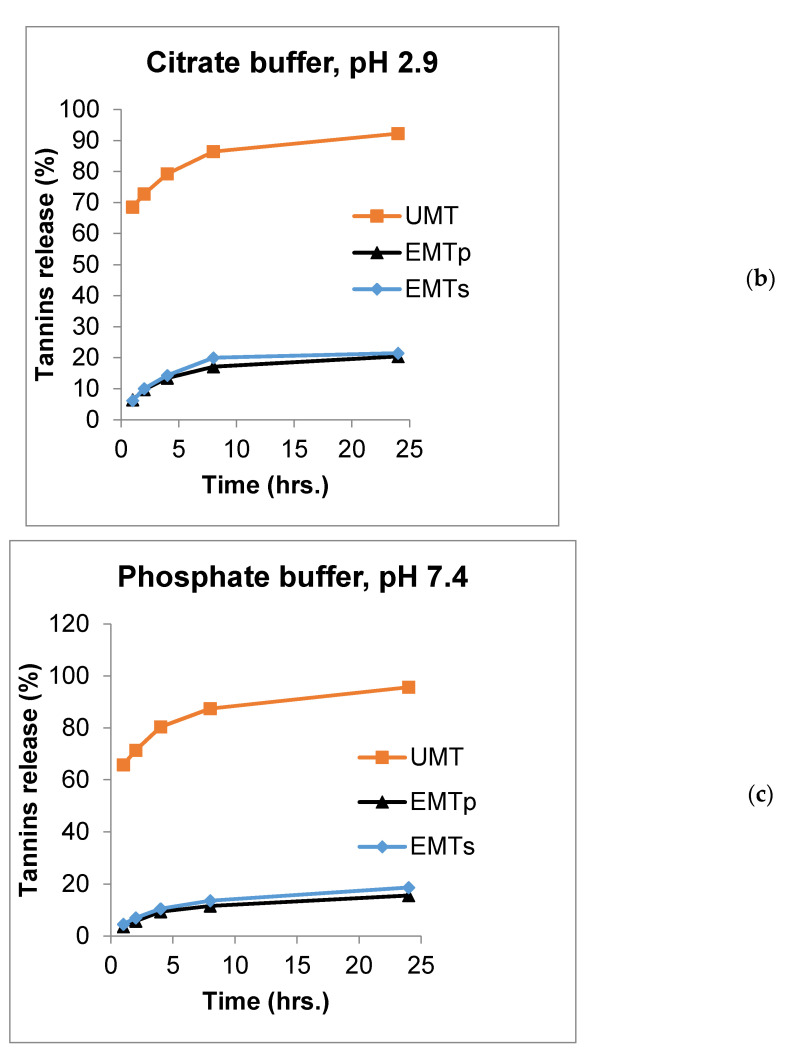
Release chart for UMT, EMT^P^ and EMT^S^ in (**a**) acetate buffer (pH 5.6), (**b**) citrate buffer (pH 2.9) and (**c**) phosphate buffer (pH 7.4).

The most exciting function of microencapsulation technology has been identified as the provision of sustained release of bioactive compounds to the targeted site at the right time which in turns improves their efficiency and bioavailability [19,40,50]. The release profiles of UMT, EMT^P^ and EMT^S^ in the rumen, abomasum and small intestine simulated buffers are displayed in Figure 2a–c. The chart shows that UMT burst rapidly in the rumen simulated buffer (pH 5.6) with 77% of the extracts released within 2 h and about 94% dissolved after a 24 h incubation period (Figure 2a). However, the EMT^P^ and EMT^S^ microcapsules eroded slowly releasing only 9% and 10% of the extracts, respectively, in the first 2 h in the acetate buffer. After 24 h, 18% of the tannins were dissolved from the EMT^P^ and 24% from the EMT^S^. Similarly, in the abomasum simulated buffer (pH 2.9), about 73% of the UMT extracts dissolved within 2 h and 92% of tannins were released after a 24 h incubation (Figure 2b). In contrast, only 10% of the extracts were released by the lipid microcapsules in the first 2 h, while 20% and 21% of the extracts were released, respectively, after a 24 h period. In the small intestine simulated buffer, 71% of the UMT dissolved in the first 2 h of incubation (Figure 2c), and after 24 h, 96% of the extracts were released in the buffer. However, only 6% and 7% were released by the EMT^P^ and EMT^S^, respectively, at 2 h. After 24 h, only 16% of the extracts were dissolved from the EMT^P^ and 19% from the EMT^S^ in the phosphate buffer. In general, the unencapsulated mimosa dissolved faster, releasing most of the extract before 24 h periods in all the buffers compared to the tannin encapsulated in sunflower oil or palm oil which dissolved slowly releasing the tannin in a sustained manner. This could be attributed to the good emulsifying properties of the palm and sunflower oils. Nevertheless, the sunflower oil microcapsule eroded faster than the palm oil microparticle which could be traced to their variations in viscosity.

The tannin release rate kinetics for the UMT, EMT^P^ and EMT^S^ in the buffers simulating the rumen, abomasum and small intestine are shown in Table 2. The results show that the release pattern for the UMT was best fitted to a negative regression model according to the First order kinetics (R^2^ = 0.87, 0.91 and 0.96), indicated by its immediate burst into the various elution media and releasing most of the tannin extract within the first 2 h of in vitro incubation. However, the EMT^P^ and EMT^S^ microcapsules did not disintegrate quickly and released smaller proportion of tannins over a 24 h period in a controlled manner while retaining most of the tannin and thus, the release of tannin fitted with high accuracy to the square root regression equation of the Higuchi order for EMT^P^ (R^2^ = 0.86, 0.89 and 0.93) and EMT^S^ (R^2^ = 0.91, 0.81 and 0.96). This shows that the unidirectional pattern in which mimosa tannins was released into the buffer solutions followed Fick’s diffusion law as noted by [48]. Similar patterns were observed by Adejoro et al. [30] and Tolve et al. [39].

**Figure 3:**
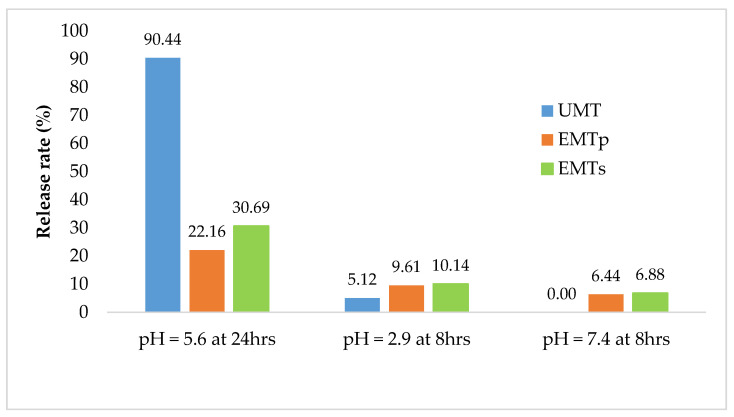
Sequential release rate for UMT, EMT^S^ and EMT^P^ in acetate, citrate and phosphate buffers.

Figure 3 presents the sequential release of tannin extract from the UMT, EMT^P^ and EMT^S^ microcapsules. In the acetate buffer, 90.4% of UMT extracts dissolved after 24 h, while in the citrate buffer 5.1% was released within 8 h but nothing was recorded in the phosphate buffer after an 8 h sequential incubation. In contrast, the EMT^S^ and EMT^P^ matrixes released 30% and 22% tannins, respectively, in the acetate buffer over the period of 24 h, and 10% each in the citrate buffer within 8 h, while in the phosphate buffer, the EMT^P^ released 6% extract and EMT^S^ 7% after an 8 h incubation. Generally, most of the extracts from the UMT were dissolved in the rumen simulated buffer and a small proportion (5%) released in the abomasum simulated buffer, while no tannin was detected in the buffer simulating the small intestine. However, for the EMT^S^ and EMT^P^, less than one third of the extracts were released in the buffer simulating the rumen after 24 h and an appreciable amount were dissolved in the abomasum and small intestine simulated media. This further confirmed the emulsifying properties of the lipid microcapsules which reduced the tannin release in the media. Literature on sequential tannin release is scarce, thus it is difficult to compare these findings with previous reports.

## 4. Conclusions

The current investigation showed that palm oil and sunflower oil could be adopted as coating materials in encapsulating Mimosa tannin to mask the tannin’s bitter taste and control the release of the extract in the GIT. The encapsulated mimosa tannins demonstrated good encapsulation efficiencies, had smaller particle sizes and were lighter than the unencapsulated tannin. Palm or sunflower oil microcapsules also stabilized the tannins’ release in gastrointestinal tract simulated buffers at predetermined periods with the potential to modify rumen fermentation. Further studies should be conducted on the lipid stability of microparticles, fatty acids transfer rate and the antioxidant properties of encapsulated tannins to justify commercial application.

## Figures and Tables

**Table 1:** In vitro release rate of unencapsulated mimosa tannin or tannins encapsulated with palm and sunflower oils in GIT simulated media.

Tannin	Rumen Simulated Buffer (pH 5.6)					Abomasum Simulated Buffer (pH 2.9)					Small Intestine Simulated Buffer (pH 7.4)				
Types	1 H	2 H	4 H	8 H	24 H	1 H	2 H	4 H	8 H	24 H	1 H	2 H	4 H	8 H	24 H
UMT (%)	68.0 ^a^	76.9 ^a^	81.0 ^a^	89.6 ^a^	94.1 ^a^	68.5 ^a^	72.8 ^a^	79.3 ^a^	86.4 ^a^	92.2 ^a^	65.8 ^a^	71.4 ^a^	80.4 ^a^	87.4 ^a^	95.7 ^a^
EMT^P^ (%)	5.19 ^b^	9.09 ^b^	12.3 ^c^	15.6 ^c^	18.3 ^c^	6.42 ^b^	9.70 ^b^	13.4 ^b^	17.0 ^b^	20.4 ^b^	3.57 ^b^	5.71 ^c^	9.34 ^b^	11.5 ^b^	15.6 ^b^
EMT^S^ (%)	6.46 ^b^	10.3 ^b^	14.8 ^b^	19.0 ^b^	23.7 ^b^	6.14 ^b^	10.0 ^b^	14.3 ^b^	20.0 ^b^	21.4 ^b^	4.47 ^b^	6.96 ^b^	10.5 ^b^	13.6 ^b^	18.6 ^b^
SEM	0.19	0.39	0.47	0.75	0.93	0.71	0.74	0.92	0.96	0.71	0.49	0.25	0.54	0.54	0.91
*p*-value	<0.01	<0.01	<0.01	<0.01	<0.01	<0.01	<0.01	<0.01	<0.01	<0.01	<0.01	<0.01	<0.01	<0.01	<0.01

Means with different superscripts across the column (*p* < 0.01). TRT = treatment; UMT = unencapsulated mimosa tannin; EMT^P^ = encapsulated mimosa tannin in palm oil; EMT^S^ = encapsulated mimosa tannin in sunflower oil; SEM = standard error of mean; H = hour.

**Table 2:** Release rate kinetics of unencapsulated mimosa tannin or encapsulated mimosa in palm and sunflower oils in GIT simulated media.

Tannin	Model	Rumen Simulated Buffer (pH 5.6)		Abomasum Simulated Buffer (pH 2.9)		Small Intestine Simulated Buffer (pH 7.4)	
UMT	Zero	y = 0.9125x + 74.781	R^2^ (0.696)	y = 0.9033x + 72.801	R^2^ (0.7752)	y = 1.1231x + 71.346	R^2^ (0.7791)
	First	y = −0.0288x + 1.412	R^2^ (0.868)	y = −0.0249x + 1.4483	R^2^ (0.9054)	y = −0.0371x + 1.4941	R^2^ (0.9595)
	Higuchi	y = 6.1422x + 66.982	R^2^ (0.842)	y = 5.9738x + 65.34	R^2^ (0.9042)	y = 7.4188x + 62.091	R^2^ (0.9067)
EMT^P^	Zero	y = 0.464x + 8.4782	R^2^ (0.712)	y = 0.5119x + 9.3987	R^2^ (0.7517)	y = 0.4521x + 5.6103	R^2^ (0.8142)
	First	y = −0.0023x + 1.9615	R^2^ (0.730)	y = −0.0033x + 1.9687	R^2^ (0.6809)	y = −0.0026x + 1.9818	R^2^ (0.7719)
	Higuchi	y = 3.1161x + 4.5304	R^2^ (0.857)	y = 3.4061x + 5.1203	R^2^ (0.8877)	y = 2.9567x + 1.9571	R^2^ (0.9286)
EMT^S^	Zero	y = 0.6368x + 9.8738	R^2^ (0.779)	y = 0.5522x + 10.07	R^2^ (0.648)	y = 0.5424x + 6.5923	R^2^ (0.8496)
	First	y = −0.0033x + 1.955	R^2^ (0.802)	y = −0.0036x + 1.9662	R^2^ (0.6318)	y = −0.0032x + 1.9785	R^2^ (0.7983)
	Higuchi	y = 4.2071x + 4.6243	R^2^ (0.907)	y = 3.7714x + 5.2192	R^2^ (0.8063)	y = 3.5169x + 2.283	R^2^ (0.9526)
